# When Genes Reveal the Truth: Alport Syndrome Mimicking Steroid-Resistant Nephrotic Syndrome

**DOI:** 10.3390/pediatric18010003

**Published:** 2025-12-19

**Authors:** John Dotis, Antonia Kondou, George Liapis, Athina Ververi, Konstantinos Kollios, Nikoleta Printza

**Affiliations:** 1 Third Department of Pediatrics, Aristotle University of Thessaloniki, Hippokration Hospital, 546 42 Thessaloniki, Greece; kkollios@auth.gr; 2 First Department of Pediatrics, Aristotle University of Thessaloniki, Hippokration Hospital, 546 42 Thessaloniki, Greece; tkondou@hotmail.com (A.K.); nprintza@gmail.com (N.P.); 3 First Pathological Anatomy Department, Laikon General Hospital, 115 27 Athens, Greece; pathology@laiko.gr; 4 Centre of Genetics for Rare Diseases, Papageorgiou Hospital, 564 03 Thessaloniki, Greece; athina.ververi@nhs.net

**Keywords:** collagen type IV alpha-3 gene, focal segmental glomerulosclerosis, steroid-resistant nephrotic syndrome, nephropathy, diagnosis

## Abstract

Τargeted genetic sequencing in a 6-year-old with steroid-resistant nephrotic syndrome and biopsy findings of focal segmental glomerulosclerosis (FSGS) revealed a novel COL4A3 pathogenic variant (p.Arg341His). Combined with electron microscopy findings of glomerular basement membrane abnormality, this led to a diagnosis of type IV collagen-related nephropathy. This case underscores the benefit of early genetic testing in presumed FSGS for prognosis and avoiding unnecessary immunosuppression in pediatric nephrotic syndrome.

## 1. Introduction

Type IV collagen-related nephropathies are a genetically diverse group of inherited kidney disorders caused by mutations in the collagen type IV alpha-3 (COL4A3), alpha-4 (COL4A4) or alpha-5 (COL4A5) genes, which encode essential components of the glomerular basement membrane (GBM) [1]. This group includes classic Alport syndrome, thin basement membrane nephropathy and less specific patterns such as focal segmental glomerulosclerosis (FSGS) [2]. Despite the classic presentation of Alport syndrome with hematuria, proteinuria, hearing loss and ocular abnormalities, atypical phenotypes are common, especially in children [3]. Some cases mimic steroid-resistant nephrotic syndrome (SRNS), without the classic extrarenal signs, leading to diagnostic uncertainty. While renal biopsy can suggest FSGS, light microscopy and immunofluorescence frequently lack specificity. Electron microscopy can provide helpful findings, such as GBM thinning or foot process effacement, but may not be definitive [4]. In such settings, genetic testing has become essential for diagnosis and therapeutic planning.

We report a case of a child with SRNS and biopsy findings of FSGS who was ultimately diagnosed with a type IV collagen-related nephropathy after identification of a rare COL4A3 variant. This case highlights the value of genetic testing in childhood SRNS, even when biopsy suggests primary FSGS, providing a comprehensive clinical, pathological and genetic characterization.

## 2. Case Presentation

A previously healthy 5-year-old girl was referred for new-onset nephrotic syndrome, characterized by generalized edema, significant proteinuria (>1 g/m^2^/day), hypoalbuminemia (1.8 g/dL) and hyperlipidemia, consistent with idiopathic nephrotic syndrome. Initial urinalysis showed no hematuria, while her renal function and blood pressure were normal. She exhibited no extrarenal signs like hearing loss or ocular issues and her family history was unremarkable for kidney disease or hearing impairment.

**Figure 1:**
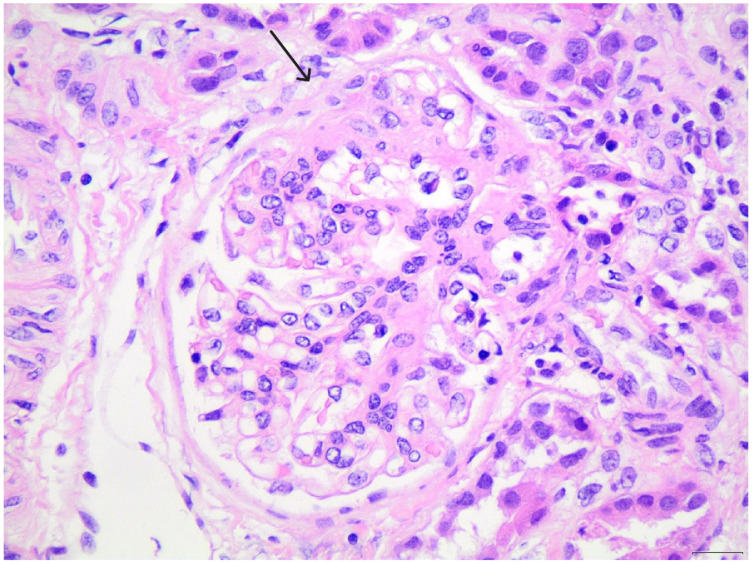
On light microscopy (H&E ×400), a small area of segmental glomerular sclerosis is visible, forming a minor synechia with Bowman’s capsule at the top of the image, as indicated by the arrow.

The patient was initially managed with high-dose oral corticosteroids (prednisolone 60 mg/m^2^/day) for 6 weeks, without achieving remission. Given the steroid-resistant course, a renal biopsy was performed (Figure 1). Approximately 30 glomeruli were present in the biopsy core, none of which were globally sclerotic, confirming adequacy of sampling. The small segmental sclerotic lesion forming a synechia with Bowman’s capsule is consistent with a perihilar variant according to the Columbia classification. Based on these light-microscopy findings, together with the presence of nephrotic-range proteinuria and the absence of hematuria or extrarenal manifestations at presentation, the patient was initially diagnosed with primary FSGS. At the time of the first biopsy, electron microscopy was not available, which further contributed to the working diagnosis. No immune complex deposition was seen on immunofluorescence microscopy.

Due to persistent nephrotic-range proteinuria and hypoalbuminemia despite an adequate course of corticosteroid therapy, a calcineurin inhibitor (cyclosporine A) was initiated based on the initial biopsy findings suggestive of FSGS. Nevertheless, the patient demonstrated only a partial and transient response, with an ongoing need for frequent intravenous albumin infusions to manage persistent hypoalbuminemia and recurrent edema, underscoring a suboptimal therapeutic effect.

Given the resistance to standard immunosuppressive treatment, a comprehensive genetic workup was initiated using a targeted next-generation sequencing panel for monogenic nephropathies. A heterozygous nucleotide substitution c.1022G>A (rs200738124) was identified in the COL4A3 gene [NM_000091.5] located on chromosome 2. This missense variant results in the replacement of arginine by histidine at amino acid position 341 (p.Arg341His) of the COL4A3 protein (Table 1). This variant is rare in the general population and has been previously reported as a variant of uncertain significance, although emerging evidence suggests potential pathogenicity in the context of collagen IV nephropathy. Repeated urine screening of both parents and the patient’s sister revealed no hematuria or proteinuria. Family-based genetic testing has been recommended but has not yet been completed.

**Figure 2:**
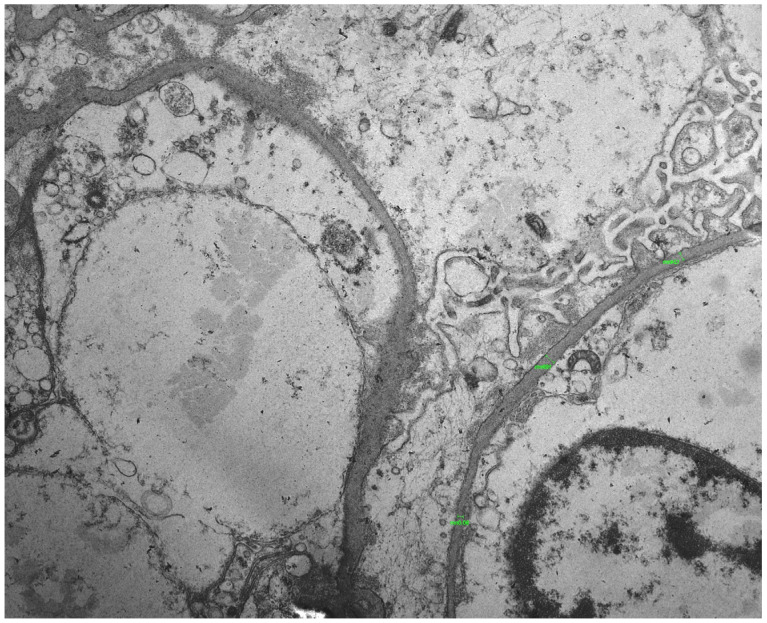
Electron microscopy (uranyl acetate, ×6000) reveals diffuse thinning of the glomerular basement membranes (GBM), with focal areas of irregular thickening and segmental lamellation. The mean GBM thickness was 153 nm, significantly reduced compared to the normal average of approximately 250 nm. This value was derived from 36 measurements across 12 GBM segments. Green markers indicate representative sites used for GBM thickness measurements. Additionally, diffuse podocyte foot process effacement was present.

Following this genetic finding, the original renal biopsy specimen was re-evaluated with electron microscopy, which had not been performed initially. Electron microscopy demonstrated focal irregular thickening and thinning of the GBM, with areas of uneven lamellation and diffuse podocyte foot process effacement involving approximately 70–80% of the capillary surface, accompanied by focal microvillous transformation, findings compatible with an underlying collagen IV-related disorder and consistent with a diffuse podocytopathy (Figure 2).

Based on the cumulative evidence from the genetic and ultrastructural analyses, a diagnosis of type IV collagen-related nephropathy was established. Immunosuppressive therapy was gradually tapered and eventually discontinued, as it was deemed unlikely to alter the natural course of the disease. Supportive therapy with the angiotensin-converting enzyme (ACE) inhibitor ramipril was continued for its antiproteinuric effect. Unfortunately, over the following months, the patient’s proteinuria remained uncontrolled and could not be maintained within the sub-nephrotic range. During this period, microscopic hematuria also began to appear in her urinalysis. This hematuria was initially intermittent but later became more consistently present, without progression, typically measuring 5–10 RBC per high-power field.

**Figure 3:**
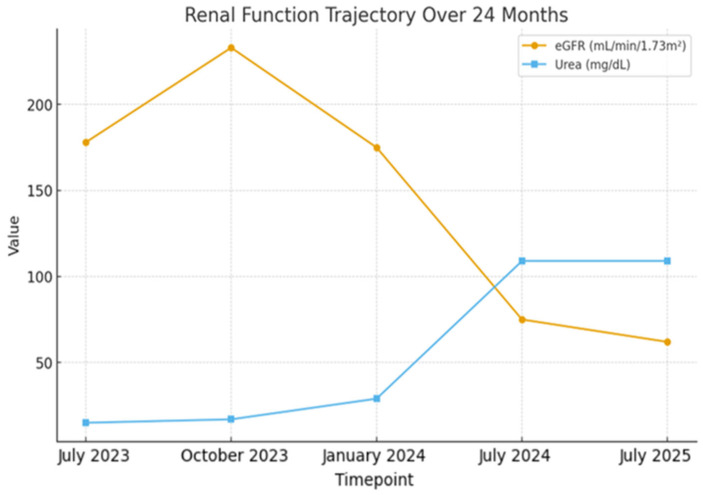
Trajectory of renal function over 24 months, showing a progressive decline in eGFR from an initially elevated value of 178 mL/min/1.73 m^2^ (consistent with hyperfiltration) to 62 mL/min/1.73 m^2^, accompanied by rising serum creatinine and urea levels.

Despite the absence of extrarenal manifestations and ongoing ACE inhibitor therapy, her renal function showed a very slow but progressive decline. Consequently, she continued to require intravenous albumin infusions every 7 to 10 days to manage persistent hypoalbuminemia and edema. Serial renal function measurements showed a gradual decline over 24 months. At presentation, her eGFR was 178 mL/min/1.73 m^2^, consistent with glomerular hyperfiltration (defined as a supraphysiologic increase in GFR relative to age-adjusted norms), and progressively decreased to 62 mL/min/1.73 m^2^, accompanied by rising creatinine and urea levels. The complete trajectory is shown in Figure 3. At the most recent evaluation, after 24 months of follow-up, the patient had not progressed to end-stage renal disease and remained in CKD stage 2–3.

## 3. Discussion

This case highlights the diagnostic complexity posed by SRNS presenting FSGS on renal biopsy, where an underlying hereditary glomerulopathy must be considered. The identification of a rare heterozygous missense variant in the COL4A3 gene (c.1022G>A; p.Arg341His), combined with ultrastructural changes observed via electron microscopy, supports the diagnosis of a type IV collagen-related nephropathy, expanding the phenotypic spectrum of collagen IV-associated kidney diseases.

Genetic testing has become a crucial component in the evaluation of children with steroid-resistant nephrotic syndrome (SRNS). Recent evidence highlights that monogenic forms account for a significant proportion of pediatric SRNS, reinforcing the need for early molecular testing in cases with atypical clinical or histological features [5].

Although FSGS is frequently considered a primary glomerular disease, it can also represent the histological manifestation of genetic disorders affecting the GBM, including mutations in COL4A3, COL4A4 or COL4A5 genes. These genes encode the α3, α4 and α5 chains of type IV collagen, a critical structural component of the GBM, cochlea and ocular basement membranes [6]. Mutations result in defective collagen networks, altering GBM integrity and function, which may be present clinically with proteinuria, hematuria and progressive renal impairment [1]. Early nephrotic-range proteinuria in heterozygous COL4A3 variants may result from impaired assembly of the α3–α4–α5 collagen IV network, leading to increased GBM permeability and heightened mechanical stress on podocytes. These alterations can promote podocyte injury and foot process effacement even before overt structural GBM changes become pronounced.

The variant detected in this patient (p.Arg341His) has been previously reported as a variant of uncertain significance in population databases and its pathogenicity remains under investigation. The absence of a family history and extrarenal manifestations in our patient aligns with the variable penetrance and expressivity often observed in heterozygous COL4A3 mutations [6,7]. This variability significantly complicates clinical diagnosis, as patients may not present with classical Alport syndrome features like sensorineural hearing loss or ocular abnormalities, especially in early childhood [8]. In a similar vein, an unusual case of X-linked Alport syndrome mimicking nephrotic syndrome has been reported, where a patient presented with FSGS findings on kidney biopsy, attributed to a likely pathogenic variant in COL4A5 [9]. Critically, the electron microscopy findings in our case provided strong pathological corroboration. They revealed irregular thickening and attenuation of the GBM, alongside diffuse podocyte foot process effacement, thus supporting a diagnosis beyond idiopathic FSGS [10].

The slow but progressive decline in renal function despite combined immunosuppressive therapy, along with the need for frequent intravenous albumin supplementation, illustrates the limited efficacy of conventional treatment in genetic forms of nephropathy [11]. This underscores the importance of distinguishing genetic collagen IV nephropathies from primary FSGS to avoid prolonged exposure to unnecessary immunosuppression. Supportive management focusing on renin-angiotensin-aldosterone system blockade with ACE inhibitors, such as ramipril, is critical for reducing intraglomerular pressure and proteinuria, thus delaying progression to end-stage kidney disease [12].

The expanding recognition of collagen IV nephropathies has led to evolving nomenclature. While Alport syndrome traditionally refers to classic phenotypes with hematuria, hearing loss and ocular anomalies predominantly in males with X-linked mutations, the broader term type IV collagen-related nephropathy better captures heterozygous autosomal forms with variable clinical presentations, including isolated proteinuria or nephrotic syndrome [2]. This terminology fosters more inclusive diagnostic and management strategies, accommodating atypical cases and those lacking extrarenal symptoms [13]. Genetic diagnosis carries significant implications for patient care and family counseling. Early identification allows for tailored surveillance of renal function, blood pressure, and extrarenal complications, as well as informed family screening to detect asymptomatic carriers or affected relatives [14]. It also facilitates avoidance of unnecessary immunosuppression and guides enrollment in clinical trials exploring novel therapies targeting the molecular pathogenesis of collagen IV nephropathies.

## 4. Conclusions

The present case emphasizes the critical role of genetic testing in children with SRNS, especially when kidney biopsy shows FSGS. The identification of a rare COL4A3 variant, supported by electron microscopy findings, led to the diagnosis of a type IV collagen-related nephropathy and justified the withdrawal of ineffective immunosuppressive treatment. Early genetic diagnosis enables precise prognosis, targeted management, and informed family counseling, while preventing unnecessary exposure to immunosuppressive therapies. Collagen IV nephropathies should be considered in the differential diagnosis of SRNS, even in the absence of classic Alport syndrome features.

## Figures and Tables

**Table 1:** Summary of the c.1022G>A (p.Arg341His) finding in the COL4A3 Gene.

Category	Element/Description	Clinical Significance and Association
Genetic finding		
Gene (OMIM)	COL4A3 (NM_000091.5, OMIM 120070), Chromosome 2	Associated with glomerular basement membrane diseases
DNA change (rsID)	c.1022G>A (rs200738124)	Substitution of guanine (G) with adenine (A) at position 1022
Protein change	p.Arg341His	Missense change: replacement of arginine (Arg) with histidine (His) at position 341
Carrier status	Heterozygous	The individual carries one mutated copy of the gene
Clinical correlation		
Associated diseases	Alport syndrome 2 (autosomal recessive, OΜΙΜ 203780), Alport syndrome 3 (autosomal dominant, OMIM 104200), Benign familial hematuria 2 (autosomal dominant, OMIM 620320)	Variable spectrum of inheritance and severity

## Data Availability

The original contributions presented in this study are included in the article. Further inquiries can be directed to the corresponding author.

## Abbreviations

The following abbreviations are used in this manuscript:

FSGS	Focal segmental glomerulosclerosis
SRNS	Steroid-resistant nephrotic syndrome
COL4A3	Collagen type IV alpha-3
COL4A4	Collagen type IV alpha-4
COL4A5	Collagen type IV alpha-5
GBM	Glomerular basement membrane
